# Enhancing Surgical Guidance: Deep Learning-Based Liver Vessel Segmentation in Real-Time Ultrasound Video Frames

**DOI:** 10.3390/cancers16213674

**Published:** 2024-10-30

**Authors:** Muhammad Awais, Mais Al Taie, Caleb S. O’Connor, Austin H. Castelo, Belkacem Acidi, Hop S. Tran Cao, Kristy K. Brock

**Affiliations:** 1 Department of Imaging Physics, The University of Texas MD Anderson Cancer Center, Houston, TX 77030, USA; m.awais0100@gmail.com (M.A.);; 2 Department of Surgical Oncology, The University of Texas MD Anderson Cancer Center, Houston, TX 77030, USAhstran@mdanderson.org (H.S.T.C.); 3 Department the of Radiation Physics, The University of Texas MD Anderson Cancer Center, Houston, TX 77030, USA

**Keywords:** liver vessel segmentation, deep learning, intraoperative ultrasound (IOUS) video frames, 2D-weighted U-Net model

## Abstract

Background/Objectives: In the field of surgical medicine, the planning and execution of liver resection procedures present formidable challenges, primarily attributable to the intricate and highly individualized nature of liver vascular anatomy. In the current surgical milieu, intraoperative ultrasonography (IOUS) has become indispensable; however, traditional 2D ultrasound imaging’s interpretability is hindered by noise and speckle artifacts. Accurate identification of critical structures for preservation during hepatectomy requires advanced surgical skills. Methods: An AI-based model that can help detect and recognize vessels including the inferior vena cava (IVC); the right (RHV), middle (MHV), and left (LVH) hepatic veins; the portal vein (PV) and its major first and second order branches the left portal vein (LPV), right portal vein (RPV), and right anterior (RAPV) and posterior (RPPV) portal veins, for real-time IOUS navigation can be of immense value in liver surgery. This research aims to advance the capabilities of IOUS-guided interventions by applying an innovative AI-based approach named the “2D-weigthed U-Net model” for the segmentation of multiple blood vessels in real-time IOUS video frames. Results: Our proposed deep learning (DL) model achieved a mean Dice score of 0.92 for IVC, 0.90 for RHV, 0.89 for MHV, 0.86 for LHV, 0.95 for PV, 0.93 for LPV, 0.84 for RPV, 0.85 for RAPV, and 0.96 for RPPV. Conclusion: In the future, this research will be extended for real-time multi-label segmentation of extended vasculature in the liver, followed by the translation of our model into the surgical suite.

## Simple Summary

In liver surgery, the complex and individualized nature of liver vascular anatomy makes planning and execution challenging. Traditional 2D intraoperative ultrasonography (IOUS) often suffers from interpretability issues due to noise and artifacts. This paper introduces an AI-based model, the “2D-weighted U-Net model,” designed to enhance real-time IOUS navigation by accurately segmenting key blood vessels, including the inferior vena cava, hepatic veins, portal vein, and its major branches. Our deep learning model demonstrated high performance, with Dice scores ranging from 0.84 to 0.96 across different vessels. This advancement promises improved precision in liver resection procedures and sets the stage for future development of real-time multi-label segmentation for broader liver vasculature.

## 1. Introduction

Liver cancer, according to data from the World Health Organization, is the fourth leading cause of cancer-related death globally, with 8 million new cases and 7 million deaths in the year 2018 alone [1]. In the current era of technological advancements, surgical resection stands out as the definitive and optimal treatment modality for primary and metastatic liver tumors [2]. Currently, ultrasonography (US) is the predominant imaging modality in the surgical workflow [3]. Intraoperative US (IOUS) provides surgeons with real-time information about the location of liver lesions in relation to the liver vasculature, allowing for adjustments in the surgical approach [4,5,6,7]. In the context of liver hypertrophy procedures such as Associating Liver Partition and Portal Vein Ligation for Staged Hepatectomy (ALPPS) and Portal Vein Embolization (PVE), the evaluation of liver regional volumes using CT imaging has proven to be a valuable tool. This imaging technique allows for precise assessment of liver growth and the planning of surgical interventions [8]. The information acquired through IOUS can have a substantial impact on surgical decision-making, leading to alterations in the surgical plan in as many as 30% of cases [9]. Nonetheless, an accurate understanding of 2D IOUS images demands not only the ability to mentally visualize the liver vessels three-dimensionally but also the capability to link this with the moving frames shown on the video display terminals. Achieving this probe-video frame orientation involves not just hand-eye synchronization but also a spatial mental map that allows for a precise understanding of the representation of a specific anatomical region on the screen and its corresponding location within the liver [10].

There is a growing focus on harnessing the potential of artificial intelligence (AI) for image recognition tasks in various domains, particularly in the field of medical imaging. AI has demonstrated its inherent capacity to autonomously conduct intricate feature analysis within medical images, thereby offering the promise of more precise and efficient diagnostic assessments [11]. Numerous intervention guidance systems have been developed to assist surgeons during various surgical procedures. Fusaglia et al. [12] proposed a method to reconstruct the liver from US images. Their approach entails a slow and controlled sweep of the US transducer over the liver, with the patient holding their breath during the procedure. Unfortunately, this technique adds extra steps to the standard intervention process, and its accuracy diminishes when the patient resumes regular breathing. Several studies [13,14] have showcased the capabilities of AI-based models to recognize hepatic tumors in US images, achieving high sensitivity and specificity. Therefore, this technology holds promise for enhancing radiologists’ performance in a range of clinical scenarios. However, the use of AI to detect and perform real-time recognition, detection, and segmentation of liver vasculature in IOUS videos has yet to be studied. Achieving this will contribute to expanding the scope of AI applications in surgical settings and has the potential to make intraoperative decision-making more efficient and effective.

To address this challenge, our research focuses on creating a real-time, highly accurate, and automatic AI-based system for the multiclass detection and recognition of vessels in IOUS videos. This research aims to bridge this gap by providing a solution that not only has the potential to enhance surgical precision and safety but also reduces the dependency on individual surgeon skills, with the goal of making liver resection procedures safer, more consistent, and accessible.

### Related Work

The US has emerged as a highly favored imaging modality in surgical and interventional medical scenarios because its positive predictive value is superior to that of other commonly used diagnostic tools. Despite its widespread application and numerous benefits, US imaging grapples with certain limitations. Specifically, limitations related to image quality, disparities in the contours of anatomical features, ambiguous boundaries within the images, the presence of shadows, and the identification of smaller anatomical structures pose difficulties for radiologists analyzing real-time liver US frames [14,15]. In addition, an oft-cited limitation of US is that it is highly operator-dependent. These challenges could potentially be resolved through the implementation of real-time AI-based segmentation algorithms. These algorithms promise precise delineation of liver structures within ultrasound images, thereby aiding radiologists in their diagnostic and interventional endeavors [16]. In the past decade, notable strides have been made in the segmentation of liver cancers within US images. One prominent approach has fused the U-Net (++) model complemented by the “atrous pyramid spatial module” [17]. However, the inherent constraints of the U-Net (++) [18] architecture impede the achievement of real-time performance, despite its commendable accuracy in tumor segmentation. Hengshuang et al. [19] presented the ICNet, which incorporates the task-specific knowledge for enhancing medical image segmentation as a pivotal aspect of improving model performance [20]. This is critical in scenarios with limited availability of annotated medical image datasets, which is a common challenge across various medical tasks. To bridge this gap and incorporate prior knowledge into segmentation models, several strategies have been proposed. One approach for histology gland segmentation involves custom loss functions tailored to enforce feature illustrations aligning with the detailed priors [21]. Similarly, loss functions in the fully connected network have been devised to incorporate shape priors in applications such as kidney segmentation [22], cardiac segmentation [23], and the subdivision of star-shaped features in membrane abnormalities [24].

Currently, the analysis and understanding of liver vasculature in the IOUS video rely on surgeon expertise. The addition of real-time liver vessel segmentation in the IOUS video will provide a tool for surgeons to understand the liver vascular structures more efficiently and effectively and for novice ultrasonographers to be better oriented. In this research, we aimed to design and test the performance of our proposed deep learning (DL) model for the real-time identification and segmentation of liver vessels within IOUS video frames.

## 2. Materials and Methods

**Figure 1:**
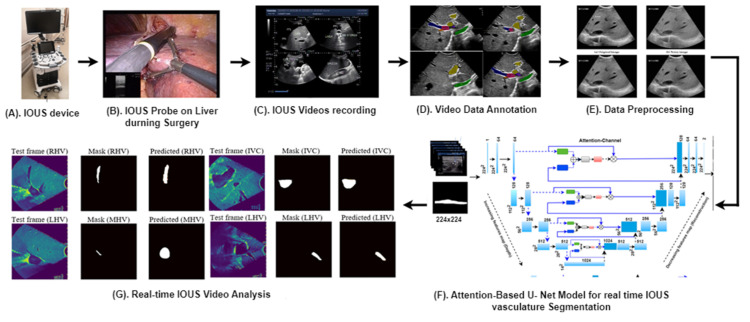
Our proposed clinical workflow and components of the AI-assisted vessel segmentation system for IOUS videos. (**A**) ARIETTA 750 intraoperative ultrasound (IOUS) device (Fujifilm, Lexington, MA, USA). (**B**) The IOUS probe positioned on the liver to analyze the internal vasculature structure. (**C**) IOUS videos captured using the Fujifilm ARIETTA 750 device and transferred to a storage system known as PACS. (**D**) Video annotation performed using RayStation (version 11B, RaySearch Laboratories, Stockholm, Sweden), where expert annotations delineate vessel regions for AI model training and testing. (**E**) Preprocessing for network input involves resizing each video frame to 256 × 256 dimensions, ensuring consistent input size for the network. (**F**) Proposed AI model, the attention-enriched U-Net, a novel AI architecture, is designed to accurately segment vessels by incorporating attention mechanisms. (**G**) Segmentation output: application of the proposed model results in vessel segmentation across multiple video frames, facilitating enhanced surgical decision-making.

Figure 1 provides an overview of our advanced methodology designed for the segmentation of multiple vessels within IOUS videos.

### 2.1. Data Preparation

This retrospective study was approved by the University of Texas MD Anderson Cancer Center Institutional Review Board (PA18-0832) involving 22 patients who underwent liver surgical procedures. Table 1 displays the demographic and medical parameters of these 22 participants. These demographic characteristics provide an overview of our study population and lay the foundation for further analysis of medical conditions and risk factors. These resections were performed by a team of experienced surgical oncologists between January 2023 and October 2023. US data were acquired using the ARIETTA 750 ultrasound device, manufactured by Fujifilm (Lexington, MA, USA). Each patient’s dataset consists of a series of US video frames, capturing various aspects of the surgical site. In total, we obtained 22 sets of IOUS videos, with each video containing 6 s/210 frames. The dataset was divided into training (60%), validation (10%), and test sets (30%) to ensure a fair evaluation of our AI system’s performance.

### 2.2. Evaluation Metrics

The model was evaluated using the following metrics: True positive (*TP*): the model’s prediction and the actual ground truth indicate the existence of positive liver vessels, aligning with each other. True negative (*TN*): both the model’s prediction and the actual ground truth align in indicating the absence of positive liver vessels. False positive (*FP*): the model’s prediction is positive, indicating the presence of liver vessels, but the actual ground truth is negative, meaning there are no positive liver vessels in that region. False negative (*FN*): the model’s prediction is negative, indicating the absence of liver vessels, but the actual ground truth is positive, meaning there are positive liver vessels in that region. These metrics are then used to compute $recall\;=\;\frac{TP}{TP\;+\;FN}$, $precision\;=\;\frac{TP}{TP\;+\;FP}$, $accuracy\;=\;\frac{TP\;+\;TN}{TP\;+\;FN\;+\;TN\;+\;FP}$, and $IOU\;=\;\frac{TP}{TP\;+\;FN\;+\;FP}.\;$ In addition, the segmentation was assessed using $Dice\_score\;=\;\frac{2\left|X\cap Y\right|}{\left|X\right|+\left|Y\right|}$ here |*X*| and |*Y*| are the cardinalities of the two predicted and actual masks.

### 2.3. Pre-Processing

**Figure 2:**
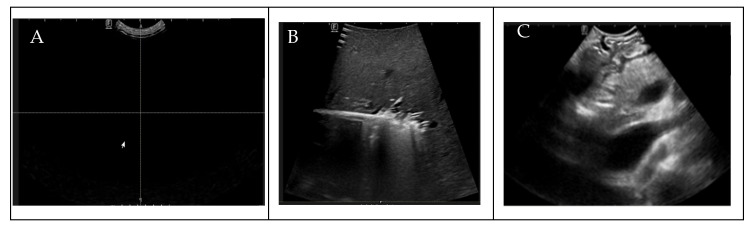
(**A**) Blackness (density ≈ 100), (**B**) no target vessels, (**C**) blurriness.

To ensure the uniformity of our dataset for DL analysis, prior to the annotation process, radiologists manually assessed the quality of the IOUS video frames before the annotation process, eliminating from the dataset that contained no anatomy within the images because of random motion of the US probe in the surgical suite and frames in which target vessels were missing, as shown in Figure 2. To train the AI model for binary vessel segmentation, each IOUS video recording was split into smaller clips based on the significant structural changes in the vasculature observed for data annotation by the radiologist. Data annotation and labeling were performed by a trained radiologist. After performing data preprocessing and segmenting the videos into smaller clips, we quantified the vessels present in each video clip, as shown in Table 2. To bolster data consistency and analytical precision, we implemented an image normalization step, adjusting the size and resolution of individual frames to create a standardized dataset for analysis.

### 2.4. Proposed Methodology

**Figure 3:**
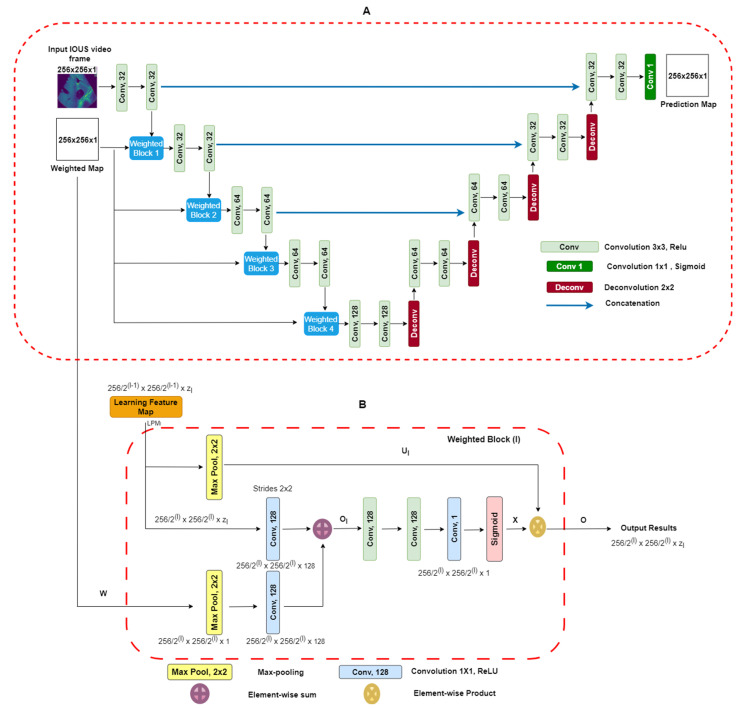
(**A**) Block diagram of proposed U-Net architecture with weighted maps. This architecture uses IOUS video frames and weighted blocks as inputs, and output segmentation predicts regions as a result. (**B**) Weighted maps l for $l\;=\;\left\{1,\ldots ,4\right\}$. We take as input the LMP values from layer L which have a spatial dimension of $\;\frac{256}{2^{l-1}}\;\times \;\frac{256}{2^{l-1}}$ along with a weighted map. The output consists of down-sampled weighted maps with a spatial dimension of $\frac{256}{2^{l}}\;\times \;\frac{256}{2^{l}}$ and includes $Z_{l}$ layers [26].

**Two-dimensional-weighted enriched U-Net model:** Our proposed architecture is built upon the AI-based U-Net model [25]. This model has demonstrated effectiveness in segmenting breast tumor ultrasound images, and we have adapted it for the task of extracting vessel structures from IOUS video data. As shown in Figure 3A, our model utilizes an attention-based segmentation approach to enhance performance. To improve its capability, the architecture features enriched attention blocks designed to precisely extract vessel structures. Following the attention-enriched layers, the network incorporates four fully connected U-Net block layers, each with a sigmoid activation function, facilitating effective segmentation of vascular regions. For robustness, dropout regularization was implemented with a fixed value of zero. This decision was made to ensure the model’s consistency and prevent overfitting during training. Overall, our architecture integrates proven methodologies to address the unique challenges presented by the IOUS vessel segmentation task.

It is imperative to note that, during model training, a strict partition was maintained to ensure that no patients from the testing dataset were present in the training dataset. This precaution was taken to eliminate data leakage and provide a fair evaluation of the model’s generalization to unseen cases. To access our model’s performance on the testing dataset, we employed the same set of statistical parameters used for the validation dataset. Compared with the U-Net model, our proposed model’s primary input comprises IOUS video frames as well as corresponding weighted maps. We adapted a weighted blocks approach to incorporate weight maps on a reduced scale throughout all layers along the contracting path of the encoder, effectively creating an image pyramid. This multi-scale approach enhances the network’s capacity to capture essential features and context from the input data. This encourages the model to concentrate on areas with elevated intensity values within the weight maps. To elaborate, the integrated weighted blocks assign increased importance to regions in the feature maps learned at each layer that display higher intensity levels in the weight maps. As a result, the specific structure and attributes of the weight maps play a decisive role in shaping the acquired feature representations. The video frames and weight maps start as 8-bit grayscale data but have been transformed into floating-point format with normalization. Both the resized video frames and weight maps are set to a standard size of 256 × 256 pixels and serve as inputs to our model. Unlike the original U-Net design, our network employs a reduced number of convolutional filters per layer, specifically (32, 32, 64, 64, 128). The network was trained for each type of liver vessel separately, recognizing the unique characteristics of each vascular structure. The liver vessels included in the study were the inferior vena cava (IVC); the right (RHV), middle (MHV), and left (LVH) hepatic veins; the portal vein (PV) and its major first and second order branches, the left portal vein (LPV), right portal vein (RPV), and right anterior (RAPV) and posterior (RPPV) portal veins, which were addressed individually to capture their distinct features and variations. The Adam optimizer, an adaptive learning rate optimization algorithm known for its efficiency, was employed during training. The learning rate was set at 0.001 to facilitate convergence and accurate gradient descent. Data augmentation was excluded during the training phase. Typical data augmentation techniques such as scaling, in-plane rotation, and translation were omitted in this study. The U-Net blocks employed during training were configured with a filter size of 3 × 3, chosen to balance between the complexity of feature extraction and computational efficiency. A batch size of 16 was chosen to ensure the optimal utilization of computational resources. The “ReLU” (rectified linear unit) activation function was employed to introduce non-linearity into the network. To further enhance the model’s learning capacity, each model was trained for 100 epochs. This training regimen allowed the model to refine its understanding of intricate vessel structures and achieve convergence, ensuring it could make accurate predictions in complex clinical scenarios. A unique feature of our approach was the use of resliced 2D vessel frames during training. Each frame was rescaled to 224 × 224 pixels to maintain uniformity. Resliced 2D frames, extracted from IOUS video data, allowed us to leverage a significant volume of original 2D IOUS frame data during training, contributing to the model’s capacity to generalize effectively. Additionally, this approach prevented overfitting, a common challenge in machine learning. By focusing on individual frames, the model was less susceptible to memorizing specific video sequences, making it more versatile and reliable in diverse clinical settings. This modification is tailored to accommodate our relatively small dataset. The probability maps, which the model generates for segmentation, maintain the same spatial dimensions as the input video frames. It is essential to note that our proposed network is trained in an end-to-end fashion. During both the training process and subsequent inference phases, the precomputed weight maps are used. **Weighted Blocks**: A visual representation of weighted block *l* is shown in Figure 3B. The learning feature map (LFM) acts as input to the weighted block, symbolized as $LFM_{L}\;=\;\left\{lfm_{1},lfm_{2},\ldots ,lfm_{z_{l}}\right\},$ Each feature map within the layer, denoted by the block at level $l\in \left\{1,2,3,4\right\}$, exhibits spatial dimensions of $\frac{256}{2^{\left(l-1\right)}}$ × $\frac{256}{2^{\left(l-1\right)}}$. The variable $z_{l,}\;$ indicates the channel dimension of the feature maps within the block *l* and takes the value of set $z_{l,}\in \left\{32,32,64,64\right\}$. Similarly, in Figure 3A, the weighted map (*w*) performs down-sampling by means of a max-pooling layer, resulting in $w_{l}$ This aligns with the dimensions of the input $LFM_{l}$ within weighted block l. Next, we use 1 × 1 convolution, followed by activation functions (ReLU), to increase the number of channels in the weighted map “W(*l*)” to “128.” An element-wise addition operation is performed between $LFM_{l}$ and $w_{l}$, leading to the creation of intermediate maps referred to as $O_{l}$. These intermediate maps have dimensions of $\frac{256}{2^{l}}\;\times \;\frac{256}{2^{l}}\;\times \;128$. Subsequently, these intermediate maps $O_{l}$ undergo further enhancement through a sequence of linear convolution with a size of 128 × 3 × 3 and 1 × 1 × 1, followed by the application of nonlinear activations (ReLU). A sigmoid activation function is used to scale and normalize the weighted maps’ values, ensuring they fall within the [0, 1] range. The resulting output is the weighted map represented as $X\;=\;\left(\partial_{y}\right)$, which has spatial dimensions $\frac{256}{2^{l}}\;\times \;\frac{256}{2^{l}}\;\times \;1$. Within this weighted map, the weighted coefficients $\partial_{y}$ are represented by assumed scalar values for each pixel y. Next, we introduce soft weights by performing element-wise products between the weighted map *X* and the max-pooled features represented as $Q_{l}$, which can be expressed ($O_{l}\;=\;X*Q_{l})$. Figure 3A visually demonstrates how this subsequent map progresses to the next layers. The design of the weighted blocks was inspired by works that employed attention gates [23,26]. In contrast to these previous models, in which weighted blocks relied on feature learning maps from hidden layers as “saliency maps” to enhance the distinctive qualities of intermediary features, we employ precomputed “saliency maps” specifically designed to pinpoint and emphasize target spatial regions. The weighted blocks used in our proposed DL model give attention to the distribution of weighted areas within the regions employed [23,26]. In numerous video frames, regions outside the vessels, representing background areas, often exhibit a degree of “saliency” in the weighted maps. To enhance segmentation outputs, we introduce additional 3 × 3 and 1 × 1 convolutional layers within the proposed weighted attention block. These supplementary layers are instrumental in refining features and, as a result, contribute to improved segmentation results. The efficacy of these additional layers was validated empirically, affirming the thoughtfully designed enhancements. **Weighted Maps:** Visual weighted estimation assumes a pivotal role within the domain of automated vasculature segmentation diagnosis, particularly when using IOUS video (Fujifilm ARIETTA 750). Its central objective lies in characterizing the importance of different regions within IOUS video frames in terms of their ability to attract the visual focus of radiologists. In the context of input video frames, our proposed system yields a visual weighted map. This map assigns weight values to individual image pixels, falling within the range of [0, 1]. Higher weight values indicate an increased likelihood that the pixel is associated with a vessel within IOUS video frames [27]. More precisely, the weighted estimation task is structured as a quadratic programming optimization. This optimization process seamlessly combines higher-level image characteristics with lower-level weighted assumptions. The architecture attributes a weighted value *W* to each super *PEL* map *y* present in an image. The architecture’s objective function is to enhance various elements, which can be broken down as follows: First, one component is a function related to a foreground map. This function calculates the probability that the region in the $y^{th}$ video frame is part of a vessel. Additionally, it considers the distance between the region represented as the $y^{\mathrm{th}}$ frame and the center of the foreground map across the IOUS video frames. In the second component of the objective function, the focus shifts to the cost associated with assigning zero saliency to a specific image region. These functions are derived from our Neutro-Connectedness methodology, which leverages insights into the degree and confidence of connectedness among image regions [27,28]. The inclusion of weighted maps in a DL model as additional prior information is based on the underlying assumption that regions in images with higher weight values are more likely to indicate the presence of tumors. Therefore, it is of utmost importance that the quality of these weight maps is adequate and provides reliable information regarding vessel locations. Otherwise, incorporating low-quality saliency maps can have a detrimental effect on the model’s performance. In our pursuit of achieving real-time segmentation of liver vessels in IOUS, we conducted a comprehensive evaluation of our model’s inference performance across different computational systems. This analysis is pivotal in understanding the practical runtime considerations before deploying the model in surgical suites.

The advantages of using weighted maps include enhanced focus on relevant regions, allowing the model to prioritize specific areas within the image that are more likely to contain vessels, thereby enhancing segmentation accuracy. Additionally, they improve performance in class imbalance scenarios by assigning greater importance to the minority class (vessels), thus improving model performance. Weighted maps also integrate prior knowledge, enhancing the model’s learning process and leading to more robust segmentations. However, there are disadvantages to consider. The effectiveness of weighted maps is contingent upon their quality; low-quality maps can mislead the model, resulting in decreased performance. Furthermore, the introduction of weighted maps complicates the training process, requiring the model to correctly interpret the additional information, which may demand more sophisticated optimization techniques and increase computational overhead. Finally, there is a risk of potential overfitting to noise in the weighted maps, which could hinder generalizability to unseen data. Therefore, it is of utmost importance that the quality of these weight maps is adequate and provides reliable information regarding vessel locations. Otherwise, incorporating low-quality saliency maps can have a detrimental effect on the model’s performance. In our pursuit of achieving real-time segmentation of liver vessels in IOUS, we conducted a comprehensive evaluation of our model’s inference performance across different computational systems. This analysis is pivotal in understanding the practical runtime considerations before deploying the model in surgical suites.

## 3. Results

**Figure 4:**
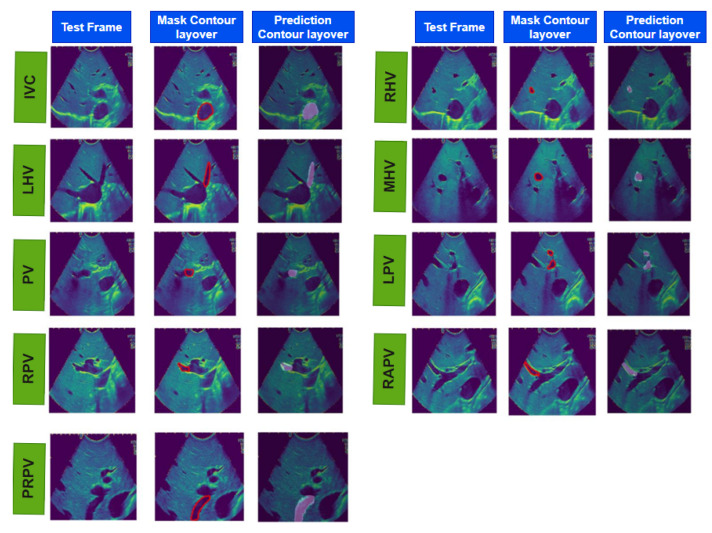
The results of real-time liver vessel segmentation on intraoperative ultrasound video frames using our proposed 2D-weighted enriched U-Net model. The figure showcases test images, mask contour layover images, and the corresponding predicted contour layover images generated by the model for multiple intraoperative ultrasound video frames.

Table 3 presents the validation performance results for our proposed model. It includes statistical parameters including the Dice score, intersection over union (IOU) score, recall, precision, accuracy, and area under the receiver operating characteristic curve (AUC–ROC) to evaluate our model’s performance in segmenting different vessels. Our proposed model achieved the highest mean Dice score of 0.93 (SD: ±0.001) for the LPV. This outcome underscores the model’s proficiency in accurately segmenting the LPV, which can have significant clinical implications. However, it is important to acknowledge that the RPV presented a more complex structural challenge. The model’s performance on RPV, as reflected by a mean Dice score of 0.84 (SD: ±0.02), was slightly lower compared with the other vessels. The intricacies and structural complexities inherent to the RPV may account for this discrepancy, as these attributes make it more challenging for our proposed model to detect the RPV with the same precision and accuracy exhibited in the Dice score for other vessels. Table 4 illustrates the performance of our model on the test dataset, and Figure 4 and Figure S1 shows the overall real-time IOUS video frames for each vessel with its input, its mask, and our model’s predicted output. Table 5 shows the comparative analysis of our model with W-Net and V-Net models. SXM2-32GB GPU. This configuration yielded even more impressive results, showcasing a runtime of less than 0.1 approx. seconds per frame. In the pursuit of optimizing real-time performance, we conducted the final round of tests on our research Kubernetes cluster using Nvidia (Santa Clara, CA, USA) DGX nodes, each equipped with 1360 A100 GPUs and a 100 Gbps Ethernet interconnect. The outcomes surpassed expectations, with a runtime of less than 0.01 approx. seconds per frame, underscoring the scalability and efficiency of our model on high-performance computing infrastructure. These systematic tests across a range of computational power systems substantiate the robustness and adaptability of our model, paving the way for its integration into the dynamic and time-sensitive environment of surgical suites.

## 4. Discussion

Our proposed AI-based 2D-weighted enriched U-Net model shows its robustness in segmenting various vessels within IOUS videos. In particular, the higher Dice scores in segmenting the multiple liver vessels in IOUS video frames show over-mode efficiency. Conversely, the challenges encountered with the lower Dice score (e.g., RPV: 0.84) underscore the nuanced complexities associated with segmenting this vessel. Our results align with recent advancements in AI-driven segmentation of vascular structures [23,25]. The achieved Dice scores, IOU, and other metrics establish our model’s competitiveness in the field. However, it is crucial to recognize that addressing structural complexities, as encountered in RPV segmentation, remains an ongoing area of research.

In the clinical context, the aggregate Dice scores attained for real-time segmentation of vasculatures in IOUS video frames signify the proficiency of the AI model in the precise delineation of critical vascular structures. These results suggest the model’s potential suitability for integration into surgical suites, thereby enhancing intraoperative decision-making and contributing to advancements in real-time image segmentation technologies.

In this study, we also compared our method, the 2D-weighted U-Net model, with well-known neural network architectures, such as U-Net, VGG16-UNet, W-Net, and V-Net, as shown in Table 5. Although these existing models have demonstrated strong performance in various applications [29,30], their training accuracy was lower than that achieved with our IOUS video frame dataset. The best training accuracy we recorded using W-Net was 0.54 for MPV, VGG16-UNet was 0.49 for LHV, and for V-Net, it was 0.54 for MHV, indicating that their performance did not meet the specific requirements of our task. The low performance of U-Net, VGG16-UNet, W-Net, and V-Net can be attributed to several technical factors. U-Net, despite its popularity in biomedical image segmentation, relies on a symmetric encoder–decoder architecture that may not effectively capture the intricate spatial relationships inherent in complex structures like liver vessels. Its use of concatenation layers to merge features from various levels can lead to suboptimal feature representation, particularly in areas with fine details. U-Net often prioritizes larger and more prominent features, which can hinder its ability to accurately identify and segment smaller vessel structures that are critical for precise segmentation, especially in datasets exhibiting significant class imbalance. VGG16-UNet, while robust in many segmentation tasks, also faces limitations due to its deep convolutional layers that may lead to overfitting, particularly when trained on limited data. This overfitting can reduce generalization capabilities, making it less effective in identifying subtle features in complex datasets like ours. W-Net, despite introducing a multi-scale approach, can suffer from training difficulties due to its complexity and potential vanishing gradient issues, which impede effective learning. This can make W-Net susceptible to noise and variability in the dataset, further complicating the segmentation of small, critical structures. V-Net, designed for volumetric data, encounters similar challenges as U-Net. Its depth can exacerbate vanishing gradient problems, limiting the model’s ability to learn from fine-grained features. Additionally, V-Net’s performance is adversely impacted by class imbalance, particularly when small vessel regions are underrepresented, causing the model to optimize primarily for the majority class and neglecting essential minority class features.

These limitations underscore the necessity for tailored architectures, such as our attention-enriched U-Net, which effectively addresses the unique challenges of liver vessel segmentation. By incorporating enhanced attention mechanisms and weighted feature maps, our proposed model significantly improves the capture of fine details and specific patterns in the segmentation task.

Our research has yielded several notable advantages, addressing the dearth of work in the domain of IOUS multivessel detection, recognition, and segmentation in video data. Currently, there exists a significant void in this area, and our research fills this gap by introducing an innovative model capable of detecting, recognizing, and segmenting liver vasculature in IOUS. This model offers a novel and essential contribution to the field of medical imaging and surgical practice. Our model has the potential to serve as a valuable surgical aid. Although the training, validation, and testing results reveal a promising Dice score and robustness of the proposed segmentation approach, when applied to real-time IOUS video frames in surgical environments, there are certain limitations. One such limitation is our model’s focus on binary segmentation, addressing individual vessels (IVC, RHV, MHV, LHV, LPV, RPV, RAPV, and RPPV) one at a time. In reality, IOUS videos often encompass multiple vessels within each frame. Therefore, an essential future direction of our research involves the expansion of a model to be capable of detecting and segmenting the diverse classes of vessels present in IOUS videos. This represents an exciting challenge and a promising avenue for improving the model’s real-world utility. One limitation of attention-based models lies in their reliance on the generation of weighted maps or masks to guide predictions. Although effective in certain contexts, these models may face challenges in real-world environments where salient features are dynamic. In our future work, we aim to develop AI-based models that are independent of specific salient features, enabling them to process real-time videos more robustly and adaptively in diverse and dynamic scenarios. Furthermore, the data used in our research were only collected from a single medical device, the Fujifilm ARIETTA 750. To ensure the robustness and generalizability of our model, future research will encompass data gathered from multiple hospitals and different devices. This diversification will allow for a more comprehensive evaluation of our model’s performance across varying real-world scenarios, making it adaptable to a broader range of clinical settings. In addition to addressing multiclass vessel segmentation, future research will also explore the detection of tumor locations within IOUS videos. Incorporating this dimension into our research is a logical progression, promising to enrich the landscape of IOUS analysis further and enhance its applicability in clinical practice. Exploring the integration of our DL model into surgical training programs represents a promising avenue for future research. This endeavor aims to enhance the educational value of our model, providing a comprehensive understanding of intricate structures within IOUS frames. Such an extension beyond immediate surgical applications can contribute to more effective and efficient training in the interpretation of vascular structures in medical images for practitioners at all levels of expertise. Additionally, our future research will also focus on integrating transformer-based architectures and unsupervised learning models. The potential of Large Language Models (LLMs) in understanding and processing contextual information presents an opportunity to enhance segmentation tasks, particularly in complex imaging scenarios. By leveraging the strengths of LLMs, we aim to improve the model’s ability to capture intricate features and relationships within the data. Moreover, exploring unsupervised learning approaches will allow us to mitigate the challenges associated with the availability of labeled datasets, enabling broader applicability of our model across various medical contexts. We are confident that these advancements will not only enhance the performance of our segmentation tasks but also contribute to the development of more robust AI solutions in medical imaging.

Overall, our research has made significant strides in the domain of IOUS video analysis, particularly in the context of multi-vessel segmentation. Its real-time capabilities offer practical utility in surgical settings, and its educational benefits extend to both surgeons and trainees. However, ongoing advancements are essential to address multi-class challenges, diversify data sources, and broaden the scope to include tumor localization. These avenues hold the promise of further enriching the landscape of IOUS analysis and expanding its applications in clinical practice.

## 5. Conclusions

In this study, we presented a DL-based approach for vessel segmentation within IOUS videos during liver surgeries. Our proposed DL model has achieved results with a mean Dice score of 0.92 for the IVC, 0.90 for the RHV, 0.89 for the MHV, 0.86 for the LHV, 0.95 for the PV, 0.93 for the LPV, 0.84 for the RPV, 0.85 for the RAPV, and 0.96 for the RPPV. It is worth noting that the model’s higher recall value compared with precision highlights a tendency to incorrectly segment structures when they are not present. This underscores the significance of expanding the dataset used for training and validation. With the inclusion of a more comprehensive dataset, we anticipate a further enhancement in the model’s performance, solidifying its potential as a robust tool for liver vessel segmentation. In the future, the integration of real-time segmentation and in-surgery implementation holds the promise of revolutionizing the field of liver surgery, emphasizing the transformative impact of DL in a clinical context. Our work represents a significant step toward improving surgical precision and patient outcomes, with the potential for broad applications in the medical field.

## Figures and Tables

**Table 1:** Patient demographic and medical parameters for 22 participants.

PID	Sex	BMI	Age, y	Pathology Report	Type of Liver Resections
1	F	24.6	66	Secondary malignant neoplasm of liver	Right Hepatectomy
2	M	25.8	47	Secondary malignant neoplasm of liver	MPS: SV, VI, VIII, anatomic Ivb
3	M	22.3	84	Secondary malignant neoplasm of liver	MPS: SVIII × 3
4	M	20.5	58	Secondary malignant neoplasm of liver	Extended left hepatectomy + Partial SV, VII, VIII
5	M	25.1	59	Intrahepatic cholangiocarcinoma	MPS: SIVa, IVb, SII
6	M	30.5	52	Secondary malignant neoplasm of liver	MPS: SII × 3
7	F	21.4	44	Secondary malignant neoplasm of liver	MPS: SVI & VII
8	F	27.9	60	Cholangiocarcinoma of the biliary tract,	Trisegmentectomy
9	F	21.2	46	Secondary malignant neoplasm of liver	MPS: SIII, Ivb
10	M	22.4	39	--	MPS: SV, VI
11	F	19.0	69	Secondary malignant neoplasm of liver.	MPS: SIII, VII, VIII
12	M	19.2	65	Secondary malignant neoplasm of liver	Partial hepatectomy SVI/VII
13	F	36.5	50	Secondary malignant neoplasm of liver	MPS: SI, II, III/IV × 2, VIII × 2, III, VI, V/VIII
14	F	20.9	75	Primary low-grade serous adenocarcinoma	MPS: SIII, IV
15	F	24.2	82	Secondary malignant neoplasm of liver	MPS: SV, II × 2 + posterior hepatectomy
16	M	27.8	69	Intrahepatic cholangiocarcinoma	Partial hepatectomy SII
17	M	32.6	48	Secondary malignant neoplasm of liver	Partial hepatectomy SII/III
18	F	21.6	56	Cholangiocarcinoma of the biliary tract	Left Lobectomy
19	M	27.4	47	Secondary malignant neoplasm of liver	Right Hepatectomy
20	F	26.9	53	Intrahepatic cholangiocarcinoma	MPS: SV, VI, VIII, anatomic Ivb
21	F	25.9	69	Secondary malignant neoplasm of liver	MPS: SVIII × 3
22	M	18.2	40	Secondary malignant neoplasm of liver	Extended left hepatectomy + Partial SV, VII, VIII

Patient ID = PID, BMI = body mass index, F = female, M = male, multiple partial resections = MPS.

**Table 2:** IOUS Video clip frame-wise database count for individual patients and for target liver vessels.

Liver Vessel	Number of Patients with Specific Vessel Types	Count of Video Clips for Specific Vessels
IVC	22	204
RHV	19	66
LHV	16	58
MHV	17	82
MPV	10	45
PRPV	8	22
RPV	11	41
LPV	15	74
ARPV	7	28

**Table 3:** Validation performance. The testing phase was conducted to assess the model’s generalization. The table reports averaged accuracy, precision scores, recall scores, Dice scores, and intersection over union (IOU) scores. The results illuminate the model’s capability to maintain robust performance across varying validation patients.

Vessels	Dice Score	IOU Score	Recall	Precision	Accuracy	AUC–ROC
IVC	0.94	0.90	0.99	0.90	0.98	0.96
RHV	0.85	0.77	0.98	0.78	0.99	0.82
LHV	0.86	0.77	0.96	0.99	0.96	0.86
MHV	0.83	0.90	0.80	0.890	0.98	0.91
MPV	0.91	0.92	0.99	0.92	0.99	0.93
PRPV	0.92	0.94	0.98	0.97	0.99	0.90
RPV	0.85	0.76	0.98	0.79	0.99	0.91
LPV	0.93	0.88	1.00	0.88	0.99	0.91
ARPV	0.86	0.77	0.99	0.77	0.98	0.87

**Table 4:** Testing dataset performance of our proposed model (mean, ± standard deviation). Statistical calculation and prediction on the testing dataset are performed after the training phase on the training dataset is completed and no further change is performed in the training script. The table reports averaged accuracy, precision scores, recall scores, Dice scores, and intersection over union (IOU) scores, revealing the model’s proficiency in vessel segmentation.

Vessels	Dice Score	IOU Score	Recall	Precision	Accuracy	AUC–ROC
IVC	0.92 ± 0.03	0.87 ± 0.02	0.94 ± 0.02	0.93 ± 0.01	0.97 ± 0.02	0.96 ± 0.02
RHV	0.90 ± 0.08	0.90 ± 0.09	0.91 ± 0.05	0.83 ± 0.08	0.99 ± 0.00	0.91 ± 0.03
LHV	0.86 ± 0.02	0.80 ± 0.03	0.90 ± 0.02	0.82 ± 0.04	0.99 ± 0.00	0.76 ± 0.03
MHV	0.89 ± 0.06	0.82 ± 0.08	0.93 ± 0.001	0.94 ± 0.03s	0.99 ± 00	0.88 ± 0.05
MPV	0.95 ± 0.05	0.91 ± 0.02	0.99 ± 0.001	0.92 ± 0.02	0.99 ± 0.001	0.92 ± 0.02
PRPV	0.96 ± 0.05	0.93 ± 0.036	0.97 ± 0.05	0.89 ± 0.05	0.99 ± 0.01	0.88 ± 0.02
RPV	0.84 ± 0.02	0.74 ± 0.04	1.00 ± 0.03	0.74 ± 0.02	0.99 ± 0.01	0.82 ± 0.05
LPV	0.93 ± 0.00	0.89 ± 0.001	0.99 ± 0.01	0.89 ± 0.03	0.99 ± 0.001	0.89 ± 0.03
ARPV	0.85 ± 0.03	0.78 ± 0.04	0.99 ± 0.00	0.78 ± 0.02	0.99 ± 0.00	0.84 ± 0.02

**Table 5:** Comparative analysis of well-known AI models in contrast to our proposed model.

DL-Model	Vessels	Training Accuracy
U-Net *	IVC	0.53
	LHV	0.51
	LPV	0.55
	MPV	0.50
	MHV	0.52
	RPV	0.53
	RHV	0.54
	ARPV	0.55
	PRPV	0.51
V-Net *	IVC	0.52
	LHV	0.51
	LPV	0.52
	MPV	0.53
	MHV	0.54
	RPV	0.48
	RHV	0.50
	ARPV	0.51
	PRPV	0.52
VGG16-UNet *	IVC	0.42
	LHV	0.49
	LPV	0.46
	MPV	0.42
	MHV	0.40
	RPV	0.42
	RHV	0.43
	ARPV	0.44
	PRPV	0.46
W-Net *	IVC	0.53
	LHV	0.52
	LPV	0.53
	MPV	0.54
	MHV	0.53
	RPV	0.49
	RHV	0.45
	ARPV	0.41
	PRPV	0.43
**Our proposed model**	IVC	0.96
	LHV	0.99
	LPV	0.92
	MPV	0.92
	MHV	0.88
	RPV	0.98
	RHV	0.86
	ARPV	0.87
	PRPV	0.81

* Max values.

## Data Availability

The data analyzed during the study are available from the corresponding author by reasonable request and in compliance with our Institutional Review Board.

## Supplementary Materials

The following supporting information can be downloaded at: https://www.mdpi.com/article/10.3390/cancers16213674/s1, Figure S1: The results of real-time liver vessel segmentation on IOUS video frames utilizing our proposed 2D-Weighted-Enriched U-Net model. The figure showcases test images, mask images, and the corresponding predicted images generated by the model for multiple intraoperative ultrasound video frames.
