# Supplementary material for: Enhancing Surgical Guidance: Deep Learning-Based Liver Vessel Segmentation in Real-Time Ultrasound Video Frames
Source: Cancers (Basel). 2024 Oct 30;16(21):3674. doi: 10.3390/cancers16213674 (PMC11545685; doi:10.3390/cancers16213674)
Supplement: Supplementary file 1 [file cancers-16-03674-s001.zip › cancers-3214463-supplementary.pdf]

Supplementary file

Figure:

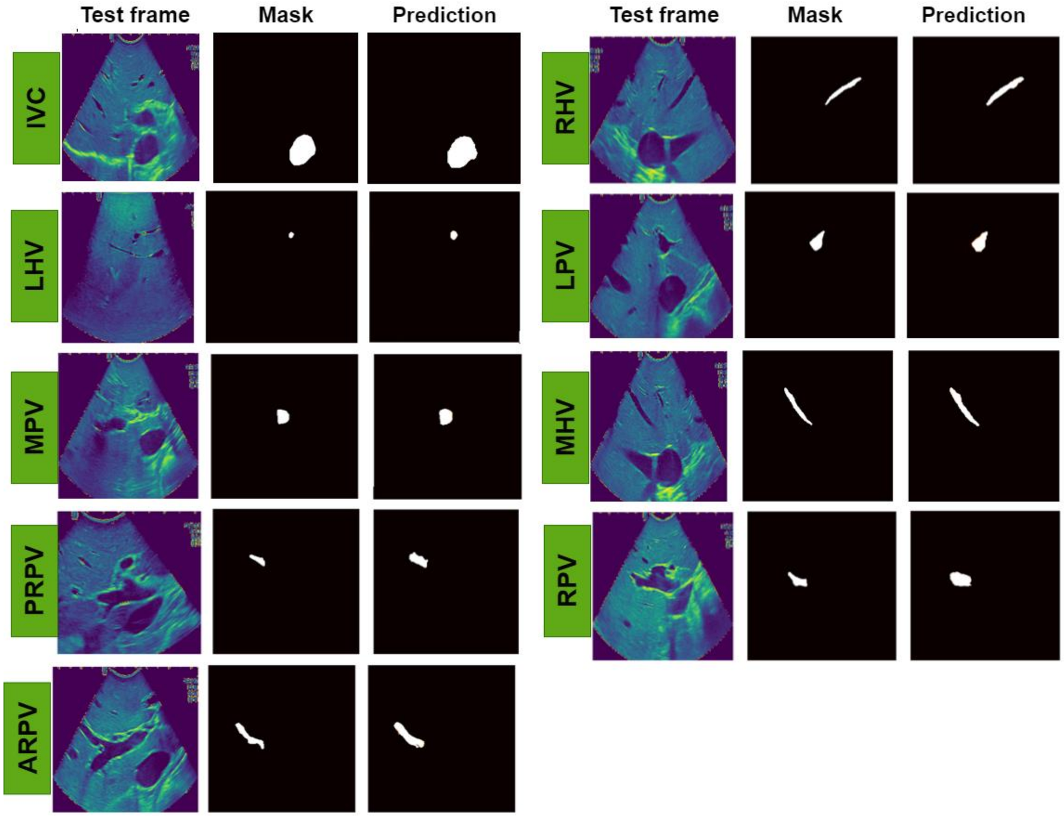

Figure S1**.** The results of real-time liver vessel segmentation on IOUS video frames utilizing our proposed 2D- Weighted-Enriched U-Net model. The figure showcases test images, mask images, and the corresponding predicted images generated by the model for multiple intraoperative ultrasound video frames
